# A Structural Context for the Mechanisms of Uncoupling Protein 1 in Brown Fat Thermogenesis

**DOI:** 10.1111/apha.70246

**Published:** 2026-05-14

**Authors:** Riccardo Cavalieri, Margeoux A. S. Dela Rosa, Camila A. Cotrim, Danielle Copeman, Mehmethan Aris, Callum Eke, Hannah Staggs‐Sandy, Paul G. Crichton

**Affiliations:** ^1^ Biomedical Research Centre, Norwich Medical School, University of East Anglia, Norwich Research Park Norwich UK

**Keywords:** brown adipose tissue, energy expenditure, fatty acid activation, mitochondrial carrier, molecular modeling, proton transport, purine nucleotide inhibition, UCP1 structure

## Abstract

Uncoupling Protein 1 (UCP1) is a defining feature of brown fat and facilitates the specialized ability of the tissue to generate heat in the process of non‐shivering thermogenesis. The protein is activated by fatty acids, which overcome its inhibition by purine nucleotides, to catalyze proton leak across the mitochondrial inner membrane, uncoupling nutrient oxidation from ATP production to release energy as heat. Thermogenesis through this process contributes to thermoregulation in many mammals and can promote nutrient turnover in humans to support metabolic health. UCP1 is a member of the mitochondrial carrier family of solute exchangers. For many years, its underlying mechanisms of activity and regulation have remained unclear. However, recent cryo‐EM structures of UCP1 have clarified details on nucleotide inhibition and, with advances in our understanding of the mitochondrial carrier transport mechanism, provided important molecular constraints to rationalize how the protein may operate. Here, we review the molecular nature of UCP1, re‐evaluating past structure–function relations in this structural context. Key carrier features and putative novel bonding that likely support state changes in the protein and proton leak activity are highlighted, as well as new hypotheses to explain subtleties in purine nucleotide binding discrimination.

**Abbreviations**
BAT: brown adipose tissue
NDPs: nucleotide di‐phosphates
NTPs: nucleotide tri‐phosphates
TTNPB: 4‐[(E)‐2‐(5,6,7,8‐Tetrahydro‐5,5,8,8‐tetramethyl‐2‐naphthalenyl)‐1‐propenyl]benzoic acid
UCP1: Uncoupling protein 1

## 1 Introduction

**FIGURE 1:**
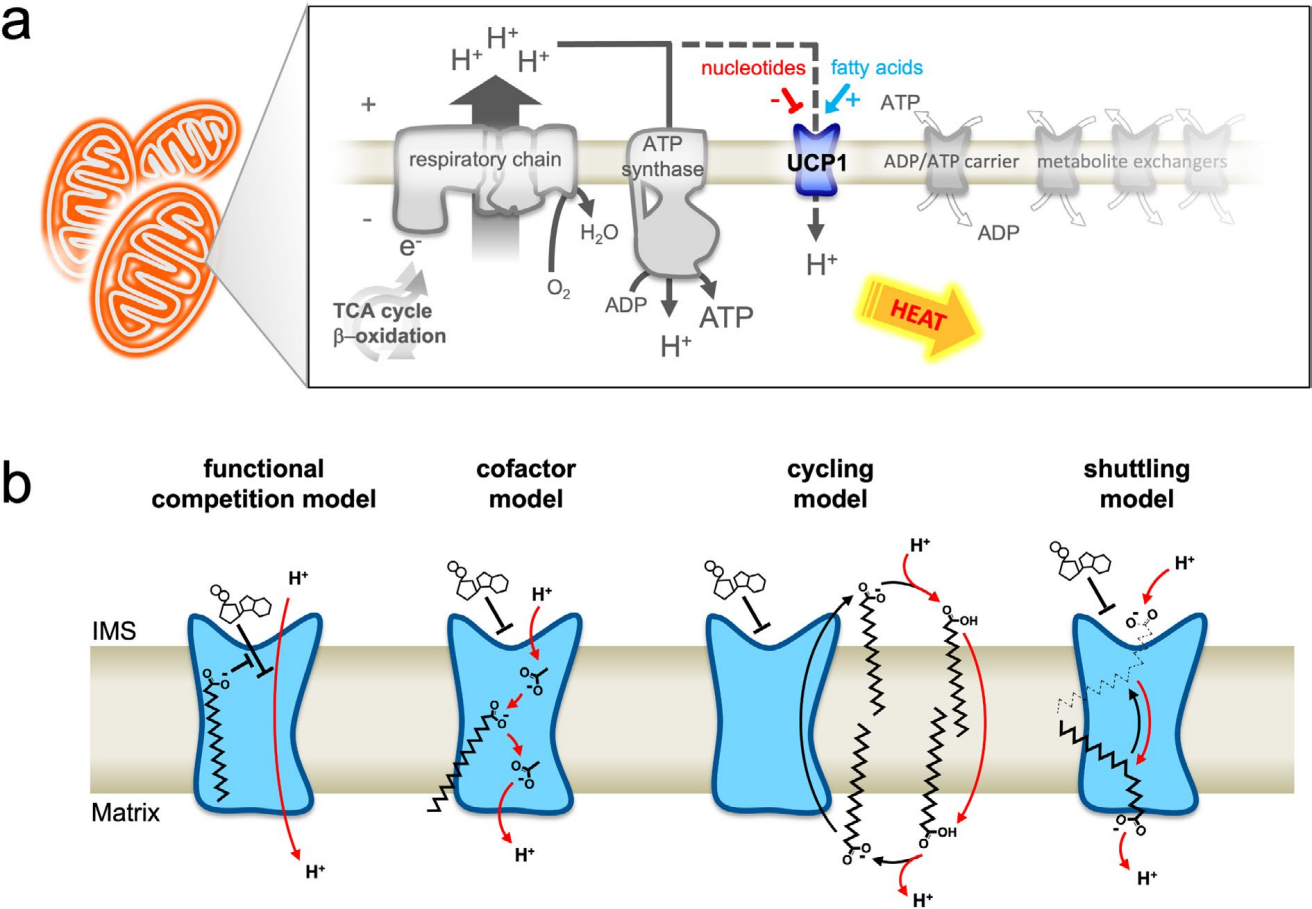
UCP1 proton leak activity in brown adipocyte mitochondria and biochemical mechanisms of ligand activation and control. (a) Activated UCP1 catalyzes the leak of protons across the mitochondrial inner membrane, dissipating the electrochemical proton gradient generated by the respiratory chain that would otherwise drive ATP synthesis. As a result, the energy from the oxidation of respiratory substrates is released as heat. Activation occurs during non‐shivering thermogenesis through adrenergic stimulation of brown adipocytes (e.g., in response to cold exposure) and release of intracellular free fatty acids, which directly interact to activate UCP1, overcoming inhibition of the protein by cytosolic nucleotides. (b) Proton transfer steps are shown by red arrows. For each model, a nucleotide molecule is depicted inhibiting from the intermembrane space (IMS) side of the membrane. Functional completion model [27]: Long chain fatty acids act merely to remove inhibiting nucleotides, either by direct competition at the nucleotide binding site or allosterically, to allow net proton transfer through the protein by an undefined process. Co‐factor model [28]: Long chain fatty acids may associate with UCP1 to provide a protonatable carboxylate group that completes a proton conductance pathway within the protein. Cycling model [29, 30]: Long chain fatty acid anions act as a transport substrate of UCP1 and are exported by the protein, flipping back across the membrane in a protonated state independently of UCP1, leading to a net proton transfer. Shuttling model [31]: Either protonated or deprotonated long chain fatty acid species bind from the cytosol and are transported by UCP1 but remain bound due to their hydrophobicity leading to only net proton transfer during transport cycling. See ref [3] for an overview.

Brown and beige adipose tissues of mammals have the ability to burn off calories as heat in the process of non‐shivering thermogenesis. These tissues are rich in mitochondria containing the integral membrane protein Uncoupling protein 1 (UCP1) that facilitates this specialized function [1, 2, 3]. When activated, the protein catalyzes the leak of protons across the mitochondrial inner membrane, dissipating the proton electrochemical gradient that would otherwise drive mitochondrial ATP production (Figure 1a). As a result, the energy from the oxidation of respiratory substrates derived from cellular nutrients is not harnessed as ATP but is instead released as heat. Thermogenesis in this manner occurs in many mammals, particularly in newborns, where it helps protect the body from cold temperatures and support thermoregulation [4]. In recent years, it has come to light that human adults may develop significant amounts of these UCP1‐containing tissues, with potential health benefits [5, 6, 7, 8]. Active human brown/beige fat correlates with leanness and better cardiometabolic health [6, 7, 8, 9, 10, 11], conferring enhanced energy expenditure, insulin sensitivity and glucose homeostasis when activated [12, 13, 14, 15]. In rodents, cold‐induced brown adipose activity can rapidly clear glucose and triglycerides from the blood [16], and loss of thermogenic activity, e.g., through knock out of the UCP1 gene, can induce obesity [17, 18]. Increasing brown/beige adipose tissue proliferation and mass, and the activity of UCP1 is a therapeutic avenue to combat metabolic disease for improved health [19].

UCP1 is not inherently active in brown adipocytes and must be switched on via acute ligand control for thermogenesis to occur. Purine nucleotides have long been known to inhibit UCP1 from the mitochondrial intermembrane space and cytosol, ATP and ADP being the most abundant [20, 21]. In response to physiological stimuli, e.g., cold exposure or overfeeding, adrenergic stimulation of adipocytes via the sympathetic nervous system induces intracellular cAMP/protein kinase A‐dependant signaling, leading to both the breakdown of cellular triglyceride stores through lipase activation to release of free fatty acids and induction of pro‐thermogenic genes as part of adaptive thermogenesis (including UCP1; see ref [22, 23]). Notably, other receptors and signaling pathways can stimulate lipolysis and partake in brown fat thermogenesis control too (see ref [24]). The free fatty acids released by lipolysis act as a fuel for mitochondrial oxidation but also as the direct activator of UCP1 proton leak, overcoming the inhibition of the protein by purine nucleotides [25, 26]. Exactly how these regulators interplay to control UCP1 activity is not well understood. However, general biochemical principles have been put forward and debated to explain how ligand interaction may facilitate and control proton leak by UCP1, each supported by different experimental approaches and observations (Figure 1b; see ref [3] for review).

Like many small eukaryotic membrane proteins, UCP1 is challenging to study and for most of > 40 years of research, the structural mechanisms underlying its activity and regulation by ligands have been somewhat of a ‘black box’. The protein is a member of the mitochondrial carrier family of solute transporters (SLC25), which are relatively unstable when purified and susceptible to incorrect folding in recombinant expression systems, increasing the risk of inaccurate conclusions on properties and function [32, 33, 34, 35, 36, 37, 38]. However, in recent years, exciting new advances have been made: firstly, in resolving structural details of the related ADP/ATP carrier in different transport states, clarifying the metabolite transport mechanism employed by mitochondrial carriers [39, 40], and, secondly, in resolving the first atomic resolution structures of UCP1 by cryo‐electron microscopy [41, 42]. These developments provide a valuable framework to rationalize the function of UCP1 and related carriers such as UCP2‐3, which, despite their name, have conventional metabolite exchanger functions distinct from UCP1 [43, 44, 45, 46], but are also important in physiology and disease [43, 47, 48, 49, 50].

Here, we give a structural overview of UCP1 and re‐evaluate past mutagenesis and functional observations in this new context to provide insights and molecular clues on how UCP1 may operate in its thermogenic functioning.

## 2 An Emerging Structural Context to Understand UCP1 Function

Mitochondrial carriers are responsible for the exchange of various small metabolites across the mitochondrial inner membrane, and all share the same core fold and membrane topology [51, 52, 53]. They function as monomers [54, 55] comprised of three repeat ~100 amino acid domains, each forming two transmembrane α‐helices with a connecting loop and short amphipathic helix towards the matrix side. Together, the three domains form a six‐transmembrane helix ‘barrel’, which surrounds a central cavity and common substrate binding region [56]. Cardiolipin lipid binds to carriers at three symmetry‐related external sites towards the matrix side, forming inter‐domain interactions [53, 57, 58, 59]. Our structural understanding of carriers has largely come from crystallography studies of the ADP/ATP carrier, which has been resolved in two different inhibitor‐stabilized states, allowing the core transport mechanism employed by the protein family to be clarified (see Ruprecht et al. [40, 60] for review). Carboxyatractyloside inhibits the ADP/ATP carrier in a ‘cytoplasmic open’ conformation (c‐state), in which the central cavity is open at the cytosolic side and closed at the matrix side by a matrix salt bridge ‘gate’ network [53, 59]. Whereas, bongkrekic acid inhibits the protein in a ‘matrix open’ conformation (m‐state), in which the central cavity is instead open to the matrix and closed at the cytoplasmic side by a cytoplasmic gate bonding network [61]. The carrier cycles between c‐state and m‐state conformations to alternate access of the central substrate binding site to either side of the membrane for stepwise substrate exchange (cf. Figure 3b). Substrate binding‐induced state shifts occur through coordinated movements in the three domains of the carrier and the breaking and reforming of the respective gate networks (see Ruprecht et al. [40, 60] for an overview).

**FIGURE 2:**
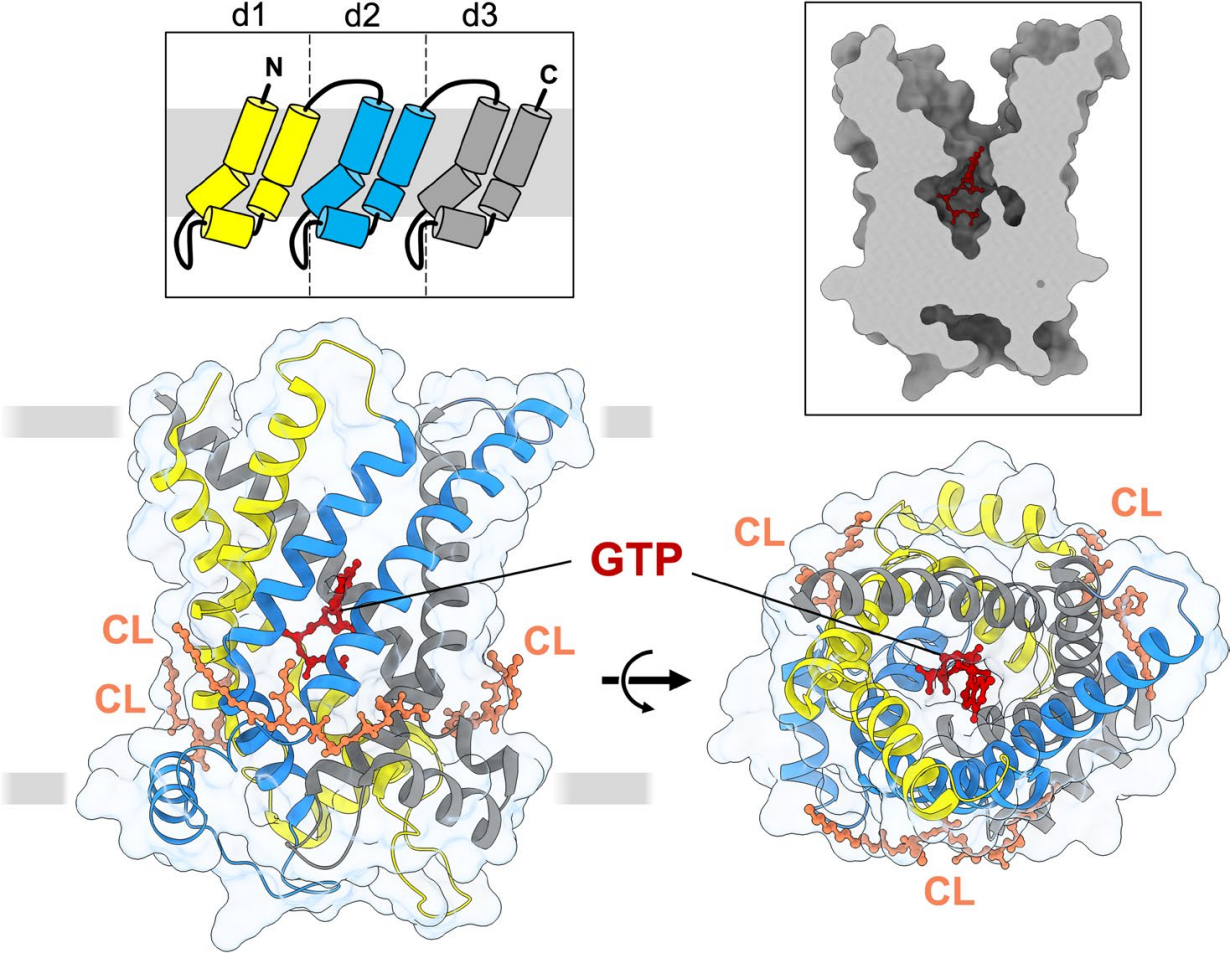
The structure of GTP‐bound human UCP1. View from the membrane (bottom left) and from the intermembrane space (bottom right) of UCP1 (PDB ID: 8G8W [41]). The three homologous domains, d1‐3 (see topology overview, top left insert), are colored yellow, blue and gray, respectively; bound cardiolipins (CL) and GTP are shown in ball and stick representation in orange and red, respectively. The structure corresponds to a c‐state of mitochondrial carriers, open at the cytoplasmic side but closed at the matrix side (see cross section view, top right insert) with GTP bound deep in the central cavity, consistent with a proton‐impermeable arrangement.

For many years, UCP1 was proposed to be mechanistically distinct from other carriers (e.g., see refs [62, 63]). However, detailed biochemical and chromatographic assessment of native UCP1 purified from lamb confirmed a typical carrier composition [64]. The protein is a monomer that binds three cardiolipin molecules, similar to other carriers, and interacts with purine nucleotides in a 1:1 stoichiometry, in contrast to early claims (see Lee et al. [64] and references therein). In 2023, the first high resolution structures of human UCP1 were determined by cryo‐electron microscopy, to reveal the protein in a GTP‐bound [41], ATP‐bound, and a non‐liganded state, as well as with bound dinitrophenol, a protonophore and mitochondrial uncoupling agent [42]. Engineered pro‐macrobody or sybody protein binding partners were used to increase the size, asymmetry, and stability of the protein and facilitate application of the approach. Each structure revealed UCP1 in a similar c‐state‐like conformation, similar to the carboxyatractyloside‐inhibited structure of the ADP/ATP carrier, with the central cavity open to the cytosol but closed at the matrix side by the matrix gate network. Purine nucleotides bind deep in the central cavity, locking the conformation, which preserves a large proton impermeable barrier, consistent with inhibition of proton leak (Figure 2). Whilst the structures have provided valuable details in how purine nucleotides bind to regulate UCP1, how fatty acids interact and induce changes to facilitate proton leak by the protein remains unclear. The similar c‐state observed in the absence of nucleotide may be a constraint of the protein binding partners used, which were all raised against the nucleotide bound complex specifically and may therefore act to fix only that conformation of the protein. Whether or not the binding of dinitrophenol, which is chemically distinct from fatty acids, in the UCP1‐sybody complex relates to physiologically relevant UCP1 activation, remains to be determined (see ref [65] for review).

## 3 The Interaction of UCP1 with Nucleotides

Mg^2+^‐free purine nucleotides (e.g., ATP, ADP, GTP and GDP) have long been known to bind at the cytosolic side of UCP1 and are believed to maintain the protein in an inhibited state in the absence or at low concentrations of activating fatty acids [20, 21]. Though recent structural studies of human UCP1 have indicated that pyrimidine nucleotides (e.g., CTP and UTP) can also bind to the protein, suggesting less selectivity than previously thought [66]. Affinity estimates of purine nucleotides (*K*_D_ values) for isolated UCP1 are in the micromolar to sub‐micromolar range [21, 64, 67], though tend to be higher for UCP1 in isolated mitochondria particularly when energized [27, 68]. The interaction displays a strong pH dependence, with high affinity observed below pH ~6.5 that drops up to 2‐orders of magnitude with increasing alkalinity towards a pH of 8 [20, 67]. Tri‐phosphate species display a higher affinity than di‐phosphate equivalents but are disproportionately sensitive to increases in pH [67]. The apparent affinity is sensitive to various other anions of salts too, which likely act as weak competitors at the binding site (e.g., phosphate, pyrophosphate, sulphate and chloride) [21, 67, 69].

**FIGURE 3:**
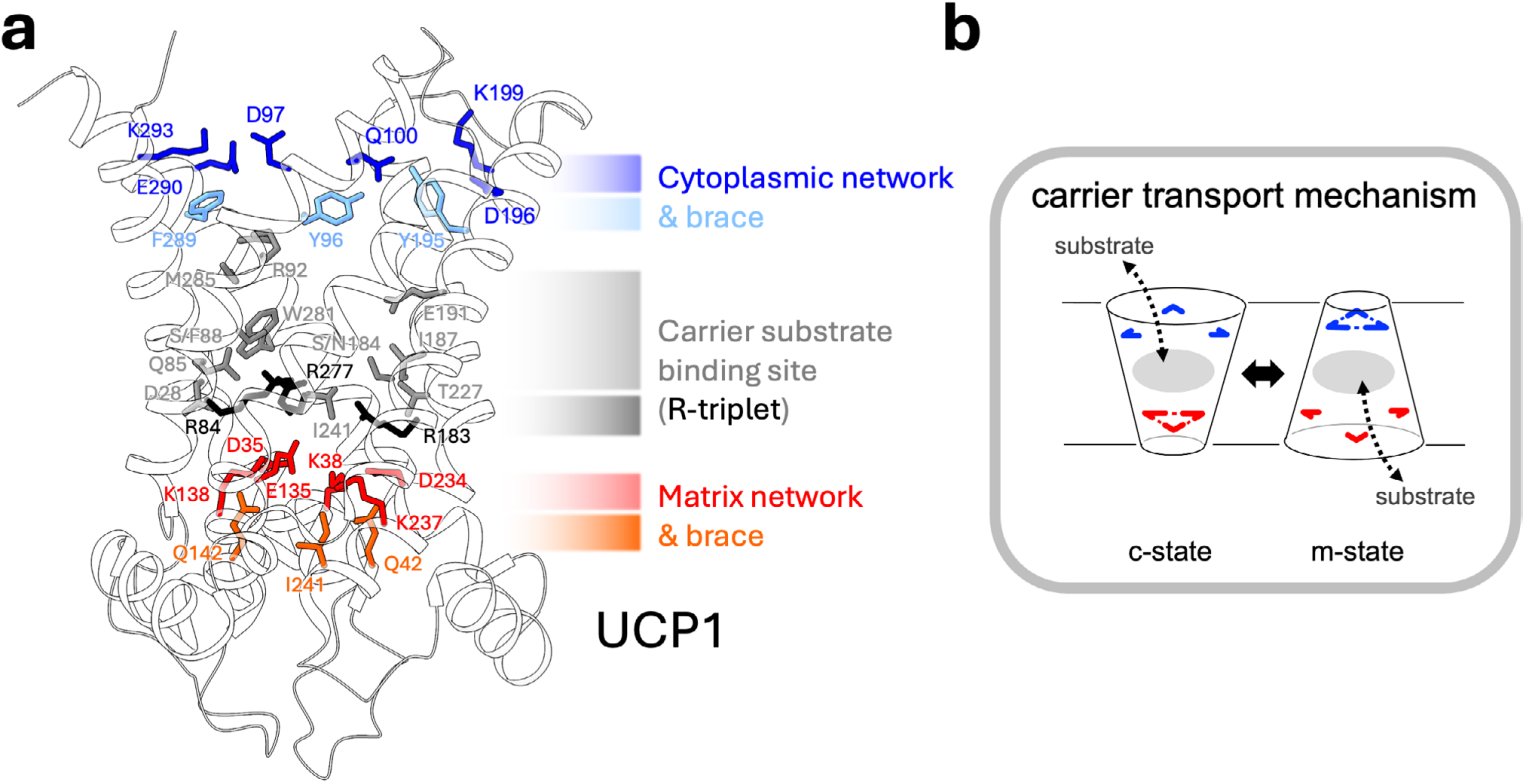
The functional elements of mitochondrial carriers in UCP1. (a) Ribbon outline of unliganded human UCP1 (PDB ID: 8HBV [42]) highlighting the amino acid residues that make up key carrier features in the transport mechanism of the ADP/ATP carrier and other carriers [60, 61]. These include residues of the cytoplasmic network (blue) and associated tyrosine braces (light blue), the central substrate binding region (gray) that contains the ‘R triplet’ (black), and the matrix network (red) with associated glutamine braces (orange). (b) The mitochondrial carrier transport process in brief summary. In a c‐state, the cytoplasmic network (blue) is open, allowing substrates from the cytosol/intermembrane space to bind in the central binding site. Binding induces the breaking of the matrix network (red) and a conformational change to the m‐state, opening access to the substrate binding site at the matrix side and closing it at the cytoplasmic side. The substrate disassociates into the matrix, freeing the binding site to accept a counter substrate for transport in the opposite direction via the reverse process (see ref [60] for details).

Cryo‐EM data on nucleotide‐bound UCP1 include GTP‐ and ATP‐bound structures [41, 42], as well as a recent UTP‐bound structure of the protein [66], all of which show a common nucleotide binding pose. Nucleotides bind in the central cavity equivalent to the common substrate binding region found in other mitochondrial carriers [70] (cf. Figures 2 and 3). The tri‐phosphate moiety orients towards the matrix side, and the base orients towards the cytoplasmic side, with multiple amino acid residues in the cavity interacting to form a tight complex. The arrangement is distinct to that likely to occur in the ADP/ATP carrier [71], consistent with nucleotides acting as inhibitors, binding only from the intermembrane space, rather than as transport substrates. The phosphates of the nucleotide form key interactions with three arginine residues, R84, R183, and R277, termed the R‐triplet, which correspond to common substrate contact points of carriers [70], as well as Q85, and K38 and K138 of the matrix salt bridge network (human UCP1 numbering). Other prominent interactions occur via the nucleotide ribose with R183 and the nucleotide base with R92, N282, N188, and E191, in which altered bonding arrangements occur for each base, potentially accounting for subtle differences in affinity observed (see ref [65, 66] for details).

The structural pose has also revealed key details to explain the characteristic pH dependence of nucleotide binding [41, 65]. As well as positively charged K38 and K138 of the matrix network, the β‐ and 𝛾‐phosphate of the nucleotide are also proximal to the negatively charged D35 and E135 of the network, which would likely repel the phosphate moiety through negative charge repulsion, weakening the interaction. However, the coordination of a proton at either (or both) phosphate would potentially facilitate proton‐mediated hydrogen bonding with either of these amino acid residues and, instead, strengthen the nucleotide interaction. Importantly, the p*K*_a_ for the protonation of the terminal phosphate of nucleotide di‐ and tri‐phosphates (NDP and NTP, respectively) is ~6.5 [72], below which the protonated form (e.g., NTP^3−^) dominates and above which the deprotonated form dominates (e.g., NTP^4−^). This value corresponds very well to the pH threshold above which UCP1 purine nucleotide affinity drops off considerably, consistent with protonation at the phosphate moiety being important for binding. Furthermore, evaluation of past UCP1 mutagenesis indicates that mutation of D35 and E135 [73, 74], or related residues in the matrix network, drastically impacts purine nucleotide affinity and the corresponding pH dependence profile, in contrast to the mutation of other residues previously implicated in pH control (e.g., E191 [75, 76, 77]), which have a far less pronounced effect.

## 4 The Interaction of UCP1 with Activators

Compared to nucleotides, the molecular nature of the fatty acid interaction is far less clear. Fatty acids and other molecules that have been demonstrated to activate UCP1 exhibit similar characteristics, which include a hydrophobic (logP > 3.5) and amphipathic nature, as well as a carboxylate head group that is sufficiently protonatable at physiological pH (p*K*_a_ > 4) [31, 78, 79]. Activator potency correlates with the size/length of the hydrophobic domain, with long chain fatty acids (e.g., 16–22 carbon) being much more potent than shorter ones [4, 28, 31], consistent with the generation of long chain species in situ from triglyceride lipolysis during brown adipose thermogenesis. Though a specific structural organization of the hydrophobic component per se appears less important [79]. Molecules with considerably varied hydrophobic regions can act as effective activators of UCP1 in vitro (e.g., retinoic acid, TTNPB, TUG‐891 and ibuprofen [78, 79, 80, 81]), which has relevance for the potential therapeutic targeting of UCP1.

The binding site for activators in UCP1 has not been established. Anions of various fatty acid analogues (e.g., alkyl sulphonates) that are not protonated at physiological pH and cannot pass directly across lipid bilayers, have been shown to be transported by UCP1 [31, 82], which has provided support for the *cycling* and *shuttling* models of how UCP1 may operate (see Figure 1b). Alkyl sulphonate transport activity is much higher than for other well‐known anion transport phenomena of UCP1 (e.g., halide transport, see ref [2]), with rates of a similar magnitude to fatty acid activated proton conductance by the protein [29, 31, 82]. Fatty acids may be expected to bind in a similar manner to transport substrates of other related carriers, interacting at the common carrier contact points composed of the R‐triplet in UCP1, and potentially compete with purine nucleotide binding. However, whilst site specific mutation of each of these arginine residues severely disrupts purine nucleotide binding, mutation appears not to compromise fatty acid‐induced proton leak [42, 83, 84]. Fatty acid binding may be less specific at these residues, with each arginine performing the same role and able to compensate for the other's loss, or binding may occur elsewhere in the protein. Notably, mutation of proximal hydrophobic residues in the central cavity (I187 and W281 to alanine) reduces inhibition by purine nucleotides but also increases the degree of stimulation of UCP1 activity induced by fatty acids [85]. The binding of fatty acid anions to the outside surface of UCP1 at the lipid‐protein interface has also been suggested (e.g., in a cycling mechanism [63]), though these regions are highly hydrophobic and lack any clear adaptations to facilitate ionic or specific hydrophobic interactions of this nature [3].

Although the binding site location has not been identified, the interaction of activators appears to induce molecular changes in UCP1 distinct from those induced by purine nucleotides. Fatty acids increase the sensitivity of UCP1 to trypsin in studies with isolated mitochondria, whereas purine nucleotides protect the protein, suggesting different conformational changes occur to either looser or tighter arrangements, respectively [68]. Additionally, thermostability analysis of purified UCP1 has indicated that, in contrast to purine nucleotides which stabilize UCP1, activators induce an ordered destabilization of the protein, consistent with a shift to a less stable state that has less underlying bonding overall [86]. Similar trends were observed in UCP1 induced by alkyl sulphonate transport substrates, as well as an equivalent influence of ADP substrate on the ADP/ATP carrier in similar conditions [32], suggesting that such shifts occur within a conventional carrier transport cycle [86].

The nature of the interplay that occurs between activators and inhibitors to control UCP1 activity during thermogenesis is not clear. In early studies with isolated mitochondria, different conductance activities associated with UCP1 in the presence or absence of regulatory ligands were clarified (S1‐4, see refs [2, 87]). At the extremes, UCP1 exhibits no proton conductance in the presence of only nucleotide (S4), consistent with structural data of the inhibited state [41, 42], and very high proton conductance in the presence of fatty acids alone, sufficient to fully abolish the protonmotive force (S1). In the absence of both ligands, a significant conductance can be observed (S2), though this likely relates to the presence of endogenous fatty acids that are not fully removed by albumin addition. Patch clamp studies of the inner mitochondrial membrane using additional approaches to remove endogenous activators have since shown that UCP1 is inactive in the absence of both ligands. In the presence of both ligands (S3), the likely physiologically relevant conditions during thermogenesis, a moderate conductance is achieved sufficient for loss of respiratory control. The millimolar concentrations of purine nucleotides in the cytosol are much higher than the *K*_D_ values that UCP1 displays for these species and would be expected to retain the protein inactive, yet fatty acids around the ~0.1 micromolar range are able to activate UCP1 in adipocytes during thermogenesis [88]. Fatty acids appear to exhibit competitive kinetics with purine nucleotides in oxygen uptake studies of isolated mitochondria [27] and in patch clamp studies of mitochondrial inner membranes [31], but do not appear to influence purine nucleotide inhibition or binding in UCP1 proteoliposome studies [28]. Whether or not fatty acids act to remove purine nucleotides from UCP1 during the process, either directly or allosterically, or if the protein can maintain alternative states that facilitate proton conductance with both ligands bound [2], remains to be determined. Other factors that potentially reduce the effective inhibitor concentration in the cytosol, such as the presence of divalent cations, Ca^2+^ and Mg^2+^, which sequester free nucleotides [89], or the degradation of cytosolic nucleotides reported to occur at thermogenesis [90], may have important roles in the regulatory interplay (see ref [2] for review).

## 5 UCP1 Retains Key Structural Elements of a Mitochondrial Carrier Transport Mechanism

The available cryo‐EM structures of UCP1 have confirmed a conventional carrier fold in the protein [41, 42] and corroborate past sequence analysis [3] to indicate that UCP1 retains key features used in a conventional mitochondrial carrier mechanism of solute exchange [40, 60]. Most prominently, these include a central substrate binding site and flanking matrix gate and cytoplasmic gate bonding networks, which are fundamental to the alternating access exchange mechanism (Figure 3). In the carrier transport process, substrate interaction at the central binding site provides the energy input to induce conformational changes (c‐ to m‐state and vice versa) and the breaking of the opposing gate, with substrate contact points acting as pivots for key helix movements [40, 60]. Hence, the relative strength of each gate network, which varies across carriers, is important for the likelihood of entering and utilizing a given state, and potentially relates to the magnitude of substate interaction that facilitates state transitions in a given direction of transport [56, 60, 91].

**FIGURE 4:**
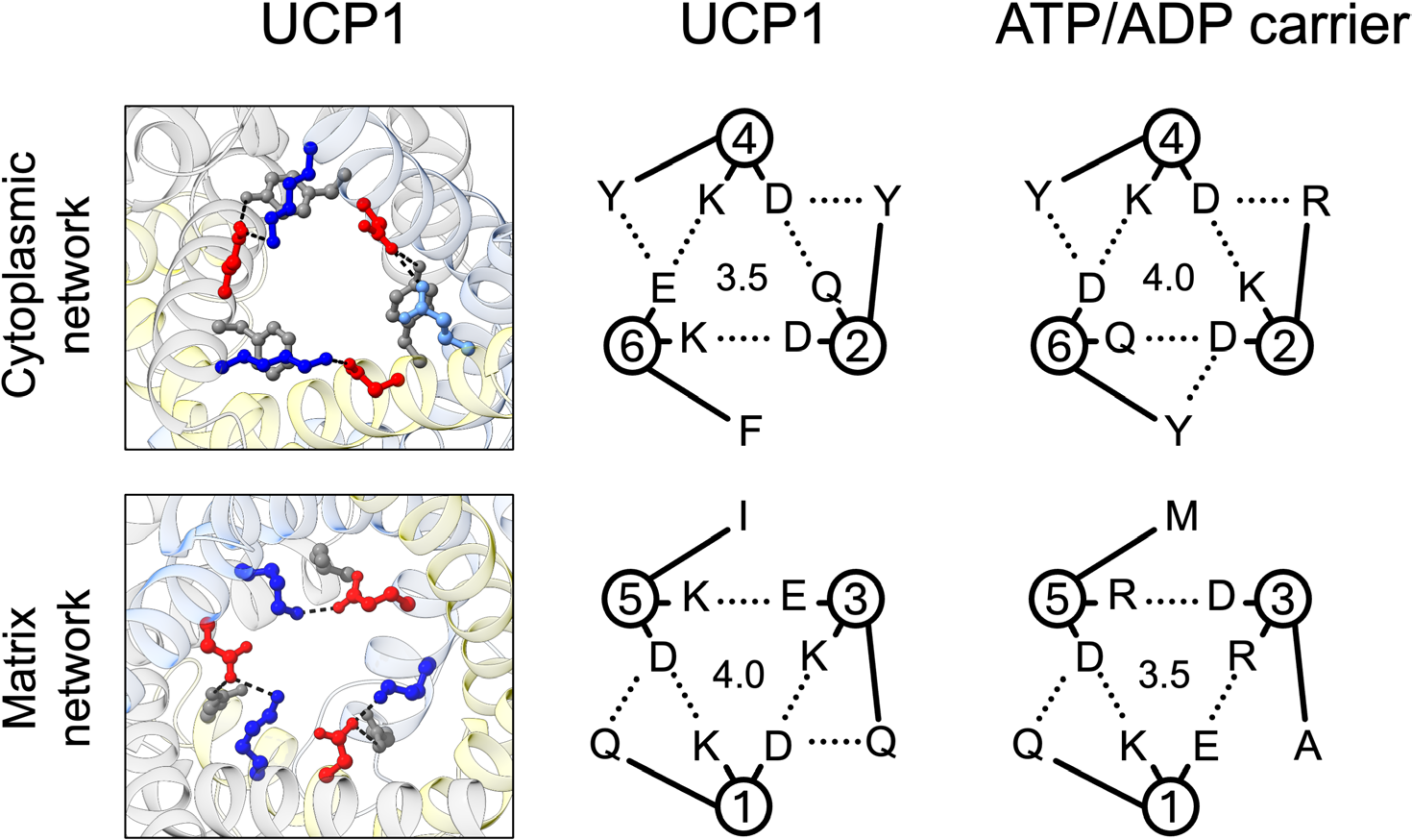
The matrix and putative cytoplasmic gate bonding networks in UCP1. The matrix network (bottom left) and putative cytoplasmic network (top left) of human UCP1, viewed from the cytoplasmic side in ball and stick representation in a c‐state structure (PDB ID: 8J1N) [42] and m‐state model of UCP1 [79] based on the structure of the ADP/ATP carrier (PDB ID: 6GCI) [61], respectively. Negatively charged, positively charged and other polar residues in the main network are shown in red, blue and cornflower blue, respectively. Residues at glutamine‐ or tyrosine‐brace positions in the matrix and cytoplasmic network, respectively, are shown in gray. Network interactions are shown as dashed lines. The corresponding bonding arrangement schemes are shown for UCP1 (middle set) and the ADP/ATP carrier (right set). Numbers in the centre give a relative estimate of the total interaction energy of the network, where salt‐bridge interactions and hydrogen bonds are 1.0 and 0.5, respectively, as outlined in ref [60].

All of the cryo‐EM structures of human UCP1 show the protein in a c‐like state, with the central cavity closed at the matrix side by residues of the matrix gate that maintains a proton impermeable barrier [41, 42, 66]. The matrix gate is composed of a primary ionic interaction network between the core elements of each domain, made up of residues from the carrier signature motif (Px[E/D]xx[K/R]), and braced further by hydrogen bonding via glutamine residues (lower left and middle panels, Figure 4). Two glutamine brace residues occur in UCP1, compared to only one found in the ADP/ATP carrier [61], giving UCP1 a slightly stronger overall matrix network bonding strength.

The conserved arginine residues of the R‐triplet in the central cavity (R84, R183 and R277) correspond to the key substrate contact points of carriers, and are similarly conserved in the related dicarboxylate and oxoglutarate carriers [70]. In the inhibited state, these residues interact with bound nucleotide (see section 3.0) and other proximal residues, whereas in the unliganded structure that lacks these contacts, they point into the central region where they could facilitate a carrier substrate‐like interaction [65]. Other residues that orient into the cavity have the potential to support binding site interactions as well, such as Q85, S/N184, N282 and D28, and S/F88, I187, W281, R92, E191 and M285 further towards the cytoplasmic side of the cavity.

In the UCP1 structures, the central cavity is open at the cytoplasmic side, consistent with a c‐state arrangement, though key residues of a [Y/F][D/E]xx[K/R] motif are present in each domain with potential to come together to form a cytoplasmic gate in a putative m‐state of the protein. UCP1 can be effectively modeled in an m‐state (e.g., refs [65, 79]), highlighting the likely bonding network (upper left and middle panels, Figure 4), composed of interdomain salt bridge interactions and hydrogen bonding via tyrosine brace residues, similar to other carriers [60]. The bonding in the primary network, supported by tyrosine braces at two of three possible positions, predicts an overall gate strength that is slightly weaker than occurs in the ADP/ATP carrier but stronger than predicted in many other carriers (Figure 4) [60]. On the inside of the gate, hydrophobic residues are present that come together to form a hydrophobic plug in the m‐state of carriers to support barrier formation [60]. UCP1 also conserves small amino acids at the interhelical interfaces of the transmembrane helices (see πGπxπG and πxxxπ carrier motifs in ref [60]), which facilitate dynamic movement and close packing of the helices in the m‐state of carriers to allow the cytoplasmic network to form. Hence, UCP1 would be predicted to use both c‐ and m‐ states and a conventional carrier mechanism for function. The relative strength predicted for both networks would also suggest a substrate interaction is needed for state shifts and transport in either direction, consistent with a strict substrate exchange process akin to the ADP/ATP carrier [56, 60, 91].

To what degree the core carrier features of UCP1 are utilized specifically for proton leak is not clear. The ability of UCP1 to transport non‐protonatable fatty acid analogues (supporting cycling and shuttling models of proton leak, Figure 1b) in this context, would imply that an underlying fatty acid transport by a conventional carrier process may be employed. The export of a fatty acid anion in exchange for protonated fatty acid, leading to net proton transfer (*shuttling* model), would align with a strict exchange process expected for comparable network strengths. Though how a long chain fatty acid, permanently bound in the process due to its hydrophobicity, as described [31], could ‘bind’ afresh and provide sufficient binding energy to break the networks in either direction is not clear. Similarly, in a fatty acid anion uniport process (*cycling* model), following export, how UCP1 would return empty to its original state without sufficient binding energy from a return substrate to break the network is also not apparent. Alternatively, it is possible the carrier features of UCP1 may have been conserved for a solute exchange function distinct from proton leak that is yet to be clarified or has been lost late in the evolutionary history of the protein (e.g., similar to the 4‐carbon metabolite exchange that UCP2 and 3 perform [43]). The observation of only a c‐state in the available cryo‐EM structures, including a ligand‐free and DNP‐bound structure, has led to a suggestion that only a c‐state is relevant to the proton leak mechanism [42, 92]. Though, as noted [65], the artificial binding partners used to increase the size, stability and asymmetry of UCP1 in cryo‐EM studies were raised against only nucleotide‐bound UCP1 in all cases and so may select and constrain the protein to a c‐state over other physiologically relevant conformations. As described in section 4.0, both trypsin sensitivity and ligand‐induced thermostability shift analysis has indicated that fatty acids induce molecular changes in UCP1 distinct from nucleotides, suggesting states beyond just one (e.g., the c‐state) are indeed relevant to activation. The similar trends induced by ADP substrate on the related ADP/ATP carrier, hint that the fatty acid‐induced changes in UCP1 are part of a conventional carrier transport cycle, potentially utilizing an m‐state. Notably, past mutagenesis of D196 in UCP1, a residue predicted to be in the cytoplasmic bonding network in a putative m‐state, is reported to induce an ~92% loss in proton leak activity (annotated as “D195N” [73]), suggesting this part of the protein has an important role in supporting activation.

## 6 Novel Bonding Interactions in a Putative m‐State?

**FIGURE 5:**
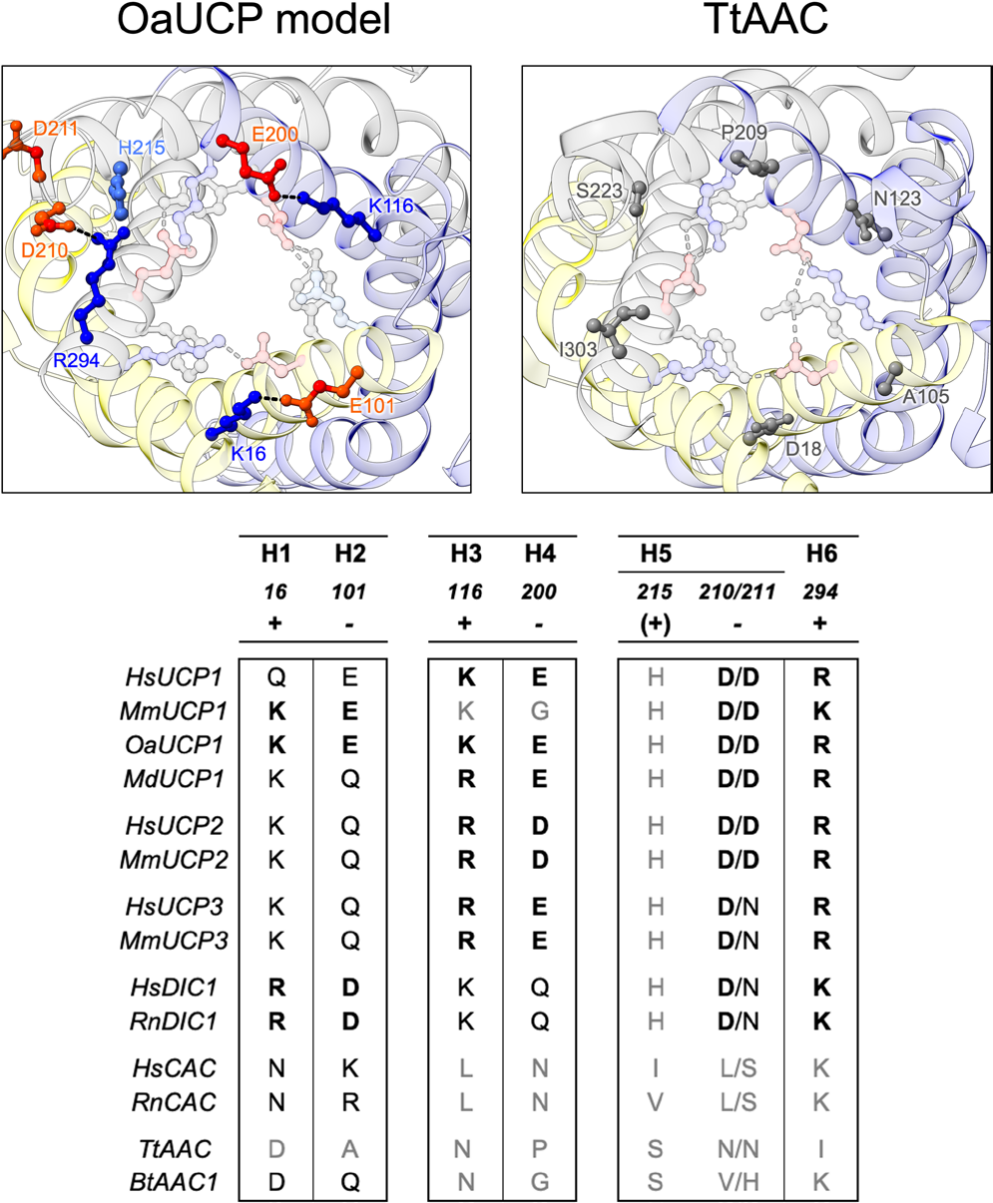
A putative secondary set of cytoplasmic network interactions in m‐state models of UCP1. Top left: View from the intermembrane space of ovine UCP1 (OaUCP1) modeled in an m‐state, highlighting residues (ball and stick representation) with potential to form a novel intra‐domain bonding network additional to the primary cytoplasmic network (human UCP1 numbering is shown). Negatively charged and positively charged residues of the secondary network are shown in red and blue, respectively, with H215 (see main text) in lighter blue. Interactions are indicated by dashed lines. The primary cytoplasmic network residues and bonding are shown in lighter pastel colors without specific labelling. Top right: Equivalent view of the ADP/ATP carrier structure (PDB ID: 6GCI [61]) that UCP1 was modeled on, lacking potential for these additional interactions. Note, Q302 is modeled in to match the wild type protein. The amino acid residues at equivalent positions are highlighted in gray. Bottom: The occurrence of equivalent residues and putative bonding in UCP1 orthologs and wider mitochondrial carriers. Amino acid residue pairs with potential to form hydrogen bond (black) or salt bridge interactions (black bold) between transmembrane helix pairs (e.g., H1‐H2) are indicated, with numbering corresponding to the position in human UCP1. Those lacking potential for an interaction are shown in gray.

In the absence of relevant structures of UCP1, we and others have modeled the protein on the m‐state structure of the ADP/ATP carrier to explore structure–function relations (e.g., see refs [65, 79, 85]). Analysis of amino acid residue interactions in modeled UCP1 highlights bonds additional to the cytoplasmic network that may occur in an m‐state of the protein but not in the c‐state [79] (Figure 5). UCP1 has the potential to form a secondary set of polar interactions at the cytoplasmic side, which are not present in the ADP/ATP carrier, that could contribute to cytoplasmic gate formation. These occur via residue pairs at equivalent positions in two of the three domains (e.g., in ovine UCP via K16‐E101 and K116‐E200 ionic interactions, Figure 5), where they bridge the two transmembrane helices within a domain, adding to the primary network that bridges helices between domains, to support gate formation. However, the equivalent residues in the third domain align positively charged R294 with H215, which may also harbor a positive charge depending on protonation state and so prevent bonding. Though other negatively charged residues, e.g., D210 or D211, occur proximal to H215 and may facilitate an ionic interaction instead. D211 was reported to be important for proton leak activity in mutagenesis studies and, along with H215 and D210, influences the pH‐dependent binding of NTPs to UCP1 without affecting the binding of NDPs [76, 77] (see below). The residues of this putative secondary cytoplasmic network are present to different degrees across UCP1 proteins and other carriers. They are present in UCP2, UCP3 and the dicarboxylate carrier (DIC), but are absent in the ADP/ATP carrier, suggesting they may represent tailoring of a general transport mechanism for carrier sub‐groups rather than be related to proton leak specifically. Though should these additional interactions occur in UCP1, they would increase the overall bonding strength of the cytoplasmic network and propensity to enter an m‐state over a c‐state.

As well as at the cytoplasmic gate, amino acid residues in the central cavity may also form m‐state‐specific interactions. Adjacent to the R‐triplet on the cytoplasmic side in the common carrier substrate binding region, hydrophobic residues occur at equivalent positions in each domain (F88, or S88 in human UCP1, I187 and W281), the latter two of which support the nucleotide interaction in the c‐state [66, 85]. These residues are separate from one another in the c‐state but would come closer together in an m‐state to form a hydrophobic patch with potential to support interactions with the fatty acid tail of activators and allow the carboxylate headgroup to form ionic interactions with the R‐triplet [79]. Surprisingly, truncating mutations (I187A or W281A) induce an *increase* in UCP1‐dependent respiratory activity in response to fatty acids [85]. Hence, these residues have an important influence on proton leak, though their exact role is not clear.

R92 and E191, which occur at equivalent positions in the first and second domain at the cytoplasmic side of the cavity, have potential to come together in an m‐state to form an ionic interaction [79]. These residues are separated in the c‐state structures, and both interact with nucleotide in the case of GTP [41, 42, 65]. Prior to structural details, R92 and E191 were implicated in nucleotide binding from mutagenesis work [75, 84]. In addition to a direct role in binding for R92, the pair were suggested to form an ionic interaction that gates access of nucleotides to the binding site in response to pH changes [89, 93]. In the current structural context, such gating may occur via formation of an m‐state, in which an R92‐E191 interaction and other m‐state specific bonding (e.g., via the cytoplasmic network) helps close the central cavity at the cytoplasmic side, blocking nucleotide access to the binding site. Recent assessment of R92E/E191R UCP1 mutants indicate a lowered fatty acid activation [85], suggesting that, whilst not essential, these residues contribute to supporting the proton leak mechanism in addition to their role in nucleotide binding.

**FIGURE 6:**
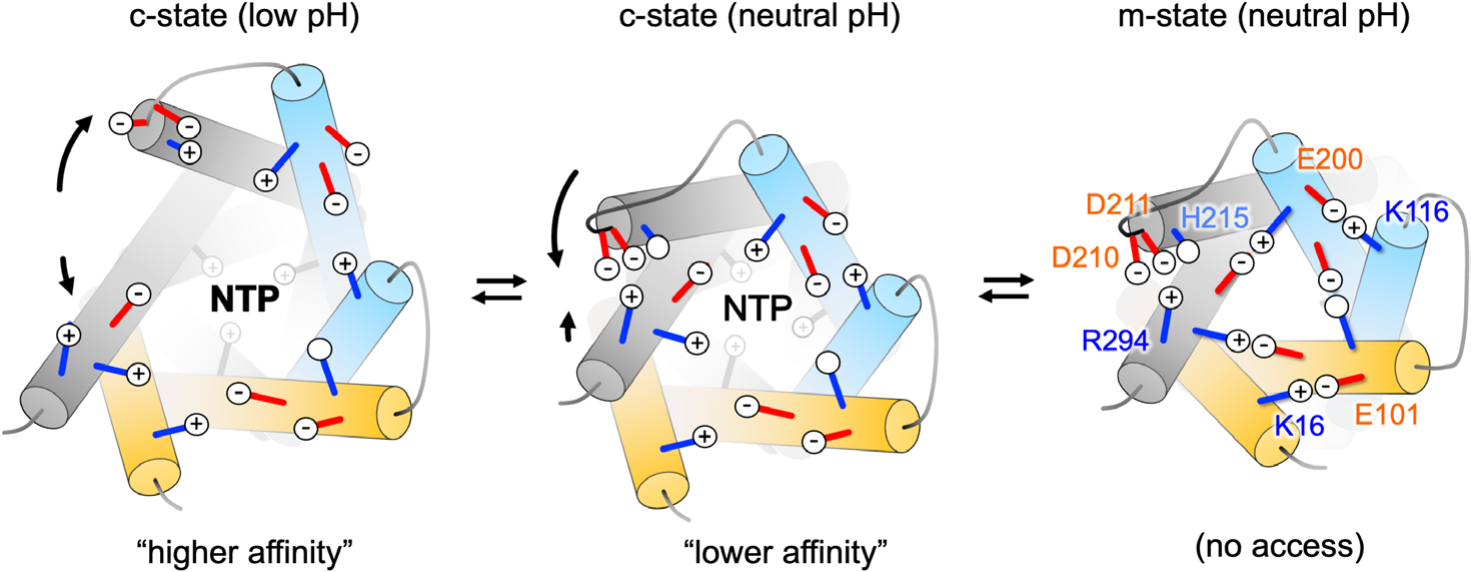
A model for how UCP1 amino acid residues that influence the binding affinity of tri‐phosphate nucleotides (NTP) specifically may act via m‐state specific bonding. View from the cytoplasmic side of UCP1 in different conformations, with the engagement state of the primary and secondary cytoplasmic network residues shown (see main text). UCP1 has the potential to transition between a nucleotide‐inaccessible m‐state (right image) and a nucleotide‐accessible c‐state. In the c‐state (at neutral pH; middle image), incomplete disengagement of the secondary cytoplasmic bonds between D210 or D211 and R294 may restrict the size of the access aperture for larger NTPs to the binding site in the central cavity, limiting the apparent affinity, without restricting smaller NDPs. H215 protonation at lower pH (< 6.6) and direct charge‐interaction with D210 would promote D210/D211‐R294 bond disengagement, fully opening the access aperture for NTPs and improving the apparent affinity (left image; see main text for further details).

Interestingly, amino acid residues potentially relevant in m‐state specific bonding correlate with those previously highlighted to influence the pH sensitivity of nucleotide affinity [79]. Mutation of E191 in UCP1 was found to dampen the pH dependence of affinity for both NDPs and NTPs [75], while mutation of H215, D210, or D211 [77], which may partake in or influence a secondary cytoplasmic network (see above), was found to dampen the profile of only the NTPs specifically (*see* Table 1 in ref [89]). As such, changes in nucleotide affinity may in part reflect the likelihood of the protein to reside in a nucleotide‐accessible c‐state over a nucleotide inaccessible m‐state, influenced by the relative strength of the underlying state‐specific bonding in each conformation [79]. These residues were previously suggested to control access and the size of the nucleotide binding pocket in earlier schemes of pH‐dependent binding [84, 89]. Although not the main driver of pH control (see section 3.0), the influence they have may be explained in the current structural context. Loss of E191 and a potential contribution to m‐state bonding via R92 may encourage net time spent in an accessible c‐state over an inaccessible m‐state resulting in the apparent increased affinity and ‘gain of function’ observed for nucleotide binding at pH ~7.5 [75]. While a reduced propensity for an m‐state at lower pH (as implied, below) could explain the contrasting *decreased* binding affinity observed at pH 6.0 [75], where a c‐state may already dominate and only direct effects of mutation on binding occur. In the case of H215, D210, and D211, these residues are proximal to one another and have the potential to restrict the size of the nucleotide access aperture in a pH sensitive manner (Figure 6). At neutral pH, an asymmetric interaction between either D211 or D210 and R294 supporting an m‐state could also be retained in a c‐state to some degree when the other symmetrical bonds of the cytoplasmic network have broken. H215 would likely be unprotonated and uncharged at this pH (average pKa of a histidine residue is ~6.6 [94]) and so would not interfere with bond formation. The resulting retention of helix 5 and 6 in close proximity at the cytoplasmic side of the protein may restrict access of larger NTP species to the main binding site in the central cavity but not smaller NDPs. At lower pH (below ~6.6), induction of a positive charge at H215 through protonation and subsequent ionic interaction with negatively charged D210 would pull both aspartic acid residues out of position, preventing the asymmetric bond. Bond disengagement and associated helix movements would allow full access of nucleotides, including the larger NTPs, and relay optimal organization of key contact points at the binding site. In such a scenario, mutation of each of these three residues and role in a common process would potentially give a similar dampening of the pH affinity profile, as has been observed [77].

## 7 Signatures of Thermogenesis

The activation mechanism of proton leak in UCP1 is unresolved, though several studies over the years have provided clues on the underlying features that are likely important for activity. Site specific mutation of several conserved amino residues in UCP1 are reported to severely reduce or abolish fatty acid‐activated proton leak. These map to various carrier features across the protein, including the matrix gate (D234 [73]), the central substrate binding region (D28 [77]), the cytoplasmic gate (D196 [73]) (and the putative secondary cytoplasmic network (D211 [77])). Some residues originally reported as essential (e.g., H146 and H148; particularly those in connecting loop regions at the matrix side of the protein), have since been found to be dispensable in separate studies on UCP1 [95, 96]. Those that appear indispensable may be important for the proton leak mechanism but could also be conserved for structural integrity and more general mechanistic roles. Of note, these residues are also conserved in UCP2 and/or UCP3, which have conventional metabolite exchange functions [43, 44] and are not thought to be thermogenic [46].

Interestingly, recent comparative biology studies have clarified the evolutionary history of brown fat and UCP1 thermogenesis, identifying the opossum UCP1 ortholog as non‐thermogenic and part of the marsupial lineage that separated before UCP1 acquired a thermogenic function in eutherian mammals [97]. Opossum UCP1 and a reconstructed ancient therian UCP1 representative do not exhibit proton leak activity, in contrast to eutherian equivalents, and likely represent conventional metabolite exchangers akin to UCP2/3. Among various amino acids altered in the eutherian UCP1s compared to the non‐thermogenic proteins, notable changes occur in the matrix and cytoplasmic gate. E135 contributes to the matrix network of thermogenic orthologs over the similarly charged D135, though it appears not to be essential for proton leak based on past mutagenesis [73, 95]. At cytoplasmic gate positions, Q100 and F289 occur in thermogenic UCP1s over K100 and Y289, respectively, giving a putative hydrogen bond instead of an ionic one, and a loss in capacity to form one of the supporting tyrosine braces, giving an apparent weaker cytoplasmic gate overall. However, scrutiny of the putative secondary cytoplasmic network suggests a compensatory change may also occur in the thermogenic proteins. E101 occurs in place of Q101, giving propensity for a stronger ionic interaction over a hydrogen bond in the network. As such, the network strength in a putative m‐state would only be marginally weaker but achieved by an altered arrangement. Exactly how the amino acid residues at these positions would influence proton leak ability is not clear, though they may serve as useful markers to predict proton leak function in UCP1 variants (with ‘Q100, E101, E135 and F289’ indicative of thermogenic activity and K100, Q101, D135 and Y289 suggesting its absence). Specific tweaks in these positions would nevertheless suggest that the gating networks, the cytoplasmic gate in particular, serve an important role in the proton leak mechanism.

## 8 Concluding Remarks

The new structural context for UCP1 has provided valuable insight into the molecular nature of this carrier at the heart of brown fat physiology and thermogenesis. Important details in how nucleotides inhibit UCP1 have been clarified, helping to explain the core inhibitory mechanism and provide a platform to understand how nucleotides and other regulators may interplay to control thermogenesis in cells. Although the activation mechanism of proton leak remains to be resolved, the structural details of UCP1 have revealed the carrier architecture and important constraints to rationalize how the protein may operate. UCP1 retains features for conventional carrier transport state changes and the potential to utilize an m‐state. Analysis on this framework highlights potential new bonding and structural hypotheses to explain the nucleotide affinity and selection behavior observed for UCP1. To what degree the mitochondrial carrier transport cycle and an m‐state in UCP1 support proton leak specifically, over other possible functions of the protein, will be important to determine along with the site of fatty acid binding, to resolve how activation occurs. Advances in our understanding of UCP1 here will inform on thermogenic energy expenditure control, as well as the nature of mitochondrial proton leak in general, and how these may be manipulated in therapeutic avenues to address metabolic dysfunction and improve health.

## Author Contributions

**Riccardo Cavalieri:** writing – review and editing. **Margeoux A. S. Dela Rosa:** writing – review and editing. **Paul G. Crichton:** writing – original draft, writing – review and editing, conceptualization. **Camila A. Cotrim:** writing – review and editing. **Mehmethan Aris:** writing – review and editing. **Danielle Copeman:** writing – review and editing. **Hannah Staggs‐Sandy:** writing – review and editing. **Callum Eke:** writing – review and editing.

## Funding

This work was supported by Biotechnology and Biological Sciences Research Council, BB/S00940X/1, BB/X017206/1.

## Ethics Statement

The authors have nothing to report.

## Conflicts of Interest

The authors declare no conflicts of interest.

## Data Availability

Data sharing not applicable to this article as no datasets were generated or analysed during the current study.
